# Quantitative Stability Evaluation of Reconstituted Azacitidine Under Clinical Storage Conditions

**DOI:** 10.3390/ph19010039

**Published:** 2025-12-23

**Authors:** Stefano Ruga, Renato Lombardi, Tonia Bocci, Michelangelo Armenise, Mara Masullo, Chiara Lamesta, Roberto Bava, Fabio Castagna, Elisa Matarese, Maria Pia Di Viesti, Annalucia Biancofiore, Giovanna Liguori, Ernesto Palma

**Affiliations:** 1 Department of Health Sciences, University of Catanzaro Magna Græcia, 88100 Catanzaro, Italy; roberto.bava@unicz.it (R.B.); fabiocastagna@unicz.it (F.C.); palma@unicz.it (E.P.); 2 Local Health Authority, ASL, 71121 Foggia, Italy; renato.lombardi@aslfg.it (R.L.); michelangelo.armenise@aslfg.it (M.A.); mara.masullo@aslfg.it (M.M.); chiara.lamesta@aslfg.it (C.L.); giovanna.liguori@aslfg.it (G.L.); 3 IRCCS Casa Sollievo Della Sofferenza, San Giovanni Rotondo (FG), 71013 Foggia, Italy; t.bocci@operapadrepio.it (T.B.); mp.diviesti@operapadrepio.it (M.P.D.V.); a.biancofiore@operapadrepio.it (A.B.); 4 University of Vita-Salute San Raffaele, 20132 Milan, Italy; e.matarese@studenti.unisr.it

**Keywords:** azacitidine (AZA), stability, HPLC, myelodysplastic syndromes, degradation

## Abstract

**Objectives**: The aim of this study was to evaluate the stability of azacitidine (AZA) under clinical storage conditions (room temperature vs. refrigeration) to identify practical protocols that minimize waste and improve cost-effectiveness. **Methods**: AZA solutions (1 mg/mL) were stored at 23 ± 2 °C or 4 °C. Stability was assessed using a validated high-performance liquid chromatography (HPLC) method. Chromatographic separation was achieved on a Hypersil ODS C_18_ column (250 mm × 4.6 mm, 5 μm) using an isocratic mobile phase of 50 mM potassium phosphate buffer (pH 7.0)-acetonitrile (98:2, *v*/*v*) at a flow rate of 1.0 mL/min, with UV detection at 245 nm and a 20 μL injection volume. The method demonstrated specificity for AZA and its main degradation product (DP), with LOD and LOQ of 12.56 μg/mL and 62.8 μg/mL, respectively. Linearity (R^2^ = 0.9928), precision (RSD% < 5 for mid/high levels), and accuracy (mean recovery 96%) were established. **Results**: Azacitidine degraded rapidly at room temperature, with >85% loss within 24 h. In contrast, refrigeration at 4 °C significantly delayed degradation, with only ~26% loss observed over the same 24 h period. Chromatographic analysis confirmed the formation of a primary degradation product (tentatively identified as the open-ring hydrolytic species N-(formylamidino)-N′-β-D-ribofuranosylurea based on its chromatographic behavior and literature data), consistent with the known hydrolytic pathway. The applied HPLC-UV method offered an optimal balance of specificity and practicality for monitoring this main degradation trend under clinical storage conditions, distinguishing it from more complex techniques used primarily for structural elucidation. **Conclusions**: The pronounced instability of reconstituted AZA underscores the critical importance of strict adherence to immediate-use protocols. Refrigeration provides only a limited stability window. Based on our kinetic data, maintaining the reconstituted solution within an acceptable degradation limit (e.g., ≤10% loss) at 4 °C would require administration within a very short timeframe, supporting current handling guidelines to ensure therapeutic efficacy and minimize economic waste.

## 1. Introduction

The treatment of hematological malignancies has been transformed by epigenetic therapies, such as DNA methyltransferase inhibitors [1,2,3]. Azacitidine (AZA), a cornerstone of this approach, is primarily used for treating myelodysplastic syndromes and acute myeloid leukemia (AML) [4,5]. Its mechanism of action, involving incorporation into DNA and inhibition of DNA methyltransferase, is well-established [6,7,8], and its structural integrity is critical for this pharmacological activity [5]. Although AZA is chemically stable in solid form, it rapidly degrades upon reconstitution in aqueous solution due to hydrolysis [9]. The hydrolytic instability is a well-known challenge shared by other nucleoside analogues, such as decitabine [10]. Stability studies, guided by international standards such as the ICH Q1A(R2) guideline, are essential to define a drug’s shelf-life. The use of validated, selective analytical methods, particularly High-Performance Liquid Chromatography (HPLC), is critical for these assessments. Reflecting its instability, the manufacturer of Vidaza^®^ (a brand of AZA) recommends immediate use after reconstitution, with a shelf-life of only up to 22 h when stored at 2–8 °C after reconstitution with refrigerated Water for Injection (WFI) [11]. While recent research employing optimized cold-chain reconstitution and specific suspension matrices has demonstrated the potential to extend the stability of azacitidine formulations significantly [9], the degradation kinetics under routine clinical storage conditions, using standard reconstitution practices, remain a critical practical concern. Research has confirmed that lower storage temperatures, such as refrigeration, can slow the degradation kinetics of AZA [12]. However, the short shelf-life of reconstituted AZA contributes significantly to pharmaceutical waste, a major economic burden in oncology care settings [13,14,15]. The need for optimized stability data is further highlighted by post-marketing studies that sometimes reveal significant discrepancies with initial manufacturer information [16]. The degradation pathway of AZA in aqueous solution is well-documented. In neutral to basic conditions, hydrolysis occurs via nucleophilic attack, leading to the opening of the triazine ring. Previous studies have employed various chromatographic techniques to characterize these kinetics, which are known to be highly dependent on pH and ionic strength.

The practical consequence of this degradation is a direct threat to clinical efficacy and safety. The degradation of AZA leads to a loss of pharmacological activity, as the molecule must retain its structural integrity to be incorporated into DNA and inhibit methyltransferase [17]. Consequently, administering a degraded solution results in subtherapeutic drug exposure, potentially compromising treatment efficacy and patient outcomes. Thus, defining the stability window is essential to guarantee that patients receive a therapeutically active and consistent dose.

While the instability of AZA is well-known, a detailed kinetic analysis under routine clinical storage conditions is lacking. Previous studies have not systematically evaluated whether simple storage modifications could reliably extend the shelf-life of AZA solutions in a practical, clinical setting. Therefore, this study aimed to quantitatively evaluate the stability of reconstituted AZA stored at room temperature (23 ± 2 °C) and under refrigeration (4 °C) to determine if such simple protocols could minimize waste and improve cost-effectiveness without compromising quality. For this purpose, HPLC with UV detection was selected as the analytical technique. While simpler methods like spectrophotometry could track the overall loss of the parent compound, they lack the specificity to distinguish AZA from its degradation products. Conversely, more complex techniques like LC-MS were not deemed necessary for the scope of this study, which focused on quantifying the main degradation trends under controlled conditions. The chosen HPLC-UV method offers an optimal balance of specificity, accuracy, and accessibility for a stability-indicating assay, allowing for the simultaneous quantification of AZA and detection of its principal degradation products. We used a proven HPLC method with a C18 column and UV detection at 245 nm to follow the degradation of AZA and detect the resulting products.

## 2. Results

### 2.1. Validation of Analytical Methods

Initially, different HPLC methods were evaluated and tested for the analysis of AZA that could fit the stability analysis of infusion solutions such as the one under investigation. The characteristics of the selected method are given below.

### 2.2. Analysis of Azacitidine

The HPLC method was adapted from the literature [10] with modifications to optimize the separation for our system. HPLC was selected as the analytical technique of choice due to its high resolution, sensitivity, and specificity, making it the most widely applied method in pharmaceutical stability studies for non-volatile and thermally unstable compounds. It is particularly suited for separating AZA from its known hydrolysis products. The optimized method resulted in a symmetric and well-defined peak for AZA with a retention time of approximately 9.2 min and no interference from the solvent front or excipients, confirming the specificity of the method for the analysis of the pharmaceutical preparation. Standard solutions for method validation were prepared in WFI (Water for Injection) due to its favorable stability profile under experimental conditions; subsequently, validation of the analytical method was carried out by analyzing the parameters of linearity, accuracy, repeatability, MDL, and QL.

Forced degradation of the AZA standard solution under thermal stress (60 °C for 2 h) was performed to confirm the method’s specificity. The analysis of the stressed sample revealed a significant decrease in the AZA peak area (retention time ~9.2 min) alongside the appearance of a well-resolved peak at a lower retention time (~6.0 min), corresponding to its main hydrolysis product. This clear separation demonstrates that the method is selective for AZA in the presence of its main thermal degradation product, making it specifically suitable for monitoring the hydrolytic degradation pathway of AZA under the studied conditions. The degradation product observed at a retention time of approximately 6.0 min is referred to as DP-6.0. While structural confirmation by orthogonal techniques (e.g., LC-MS) was not performed in this study, the chromatographic behavior and retention time of DP-6.0 are consistent with the primary hydrolytic degradation product of AZA described in the literature, namely N-(formylamidino)-N′-β-D-ribofuranosylurea, which results from the nucleophilic opening of the triazine ring [9]. 

### 2.3. Validation Parameters of the Analysis Method

The HPLC method was developed and validated for the purpose of monitoring AZA degradation. Linearity was evaluated over a concentration range of 93.8 to 750.0 μg/mL, yielding a correlation coefficient (R^2^) of 0.9928. The precision of the method, expressed as relative standard deviation (RSD%), was determined at multiple concentration levels. At the lowest concentration level (near the LOQ), the RSD was 9.76%. Although this value slightly exceeds the typical threshold for full quantitative validation, it was considered acceptable for the stability-indicating purpose of the method, as the assay was primarily intended to monitor relative degradation trends of AZA over time rather than to perform absolute quantification at the lowest concentration extremes. The method demonstrated fit-for-purpose performance for tracking the main degradation pathway under investigation. More importantly, at the mid- and high-level standards, which are representative of the concentrations measured throughout the stability study, the RSD values were below 5%, demonstrating suitable precision for the intended purpose (Table 1).

The accuracy, determined as the mean recovery rate, was 96%. The limit of detection (LOD) and limit of quantitation (LOQ) were calculated to be 12.56 μg/mL and 62.8 μg/mL, respectively. These parameters indicated that the method was suitable for the qualitative and semi-quantitative tracking of AZA degradation in model solutions for this stability study. This confirms the method’s suitability for detecting and quantifying low levels of AZA and its potential degradation products (Table 1).

Taken together, these validation parameters confirm that the method is fit for its intended purpose. It demonstrates adequate specificity, linearity, accuracy, and sensitivity for monitoring the relative degradation trends of AZA in this stability study. The precision was suitable for mid- and high-concentration levels, which are most relevant for the stability assessment [18].

### 2.4. Storage and Stability

**Figure 1:**
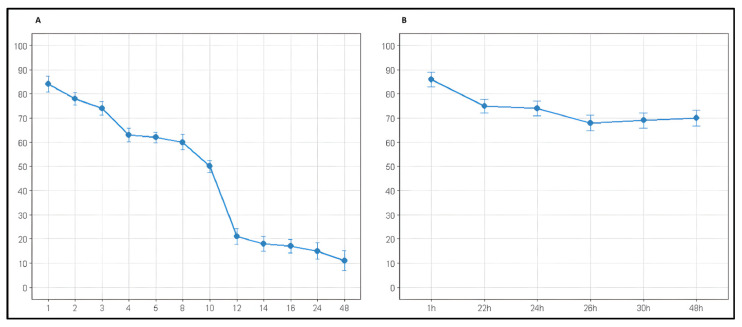
Stability of Azacitidine reconstituted and stored. (**A**) 23 ± 2 °C; (**B**) 4 °C.

The stability of AZA model solutions (1 mg/mL) was quantitatively monitored under two storage conditions. At room temperature (23 ± 2 °C), more than 15% of the drug was already lost within the first hour (84 ± 3.3% remaining). Degradation proceeded rapidly, reaching 50% loss by 10 h and leaving only 15 ± 3.5% of intact azacitidine at 24 h (Table 2, Figure 1A). Refrigeration at 4 °C significantly slowed the process: the first measurement, performed approximately 1 h after reconstitution, showed 86 ± 3.1% remaining (i.e., ~14% loss). At 24 h, 74 ± 3.0% of the drug was still present, corresponding to an overall loss of approximately 26% (Table 3, Figure 1B). Degradation appeared to slow further after 24–30 h, with ~70% remaining at 48 h.

Linear regression analysis quantified the significant protective effect of refrigeration. At 23 °C, AZA degraded at −1.64 ± 0.42%/hour (R^2^ = 0.603, *p* < 0.01), while at 4 °C the degradation rate was 4.6-fold slower at −0.358 ± 0.120%/hour (R^2^ = 0.689, *p* < 0.05). This demonstrates that refrigeration substantially delays AZA degradation while not completely preventing it.

**Figure 2:**
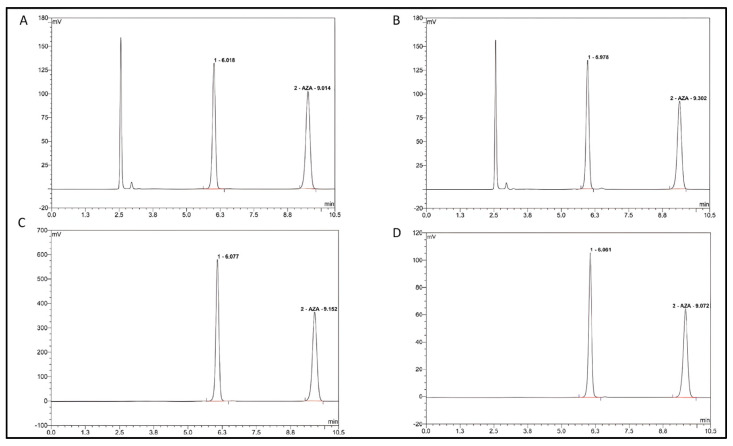
Stability of Azacitidine over time. (**A**): 3 h after reconstitution, (**B**): 5 h after reconstitution, (**C**): 8 h after reconstitution, (**D**): 10 h after reconstitution. The red line beneath the peaks represents the baseline integrated by the chromatographic software, used for accurate peak area integration and quantification.

**Figure 3:**
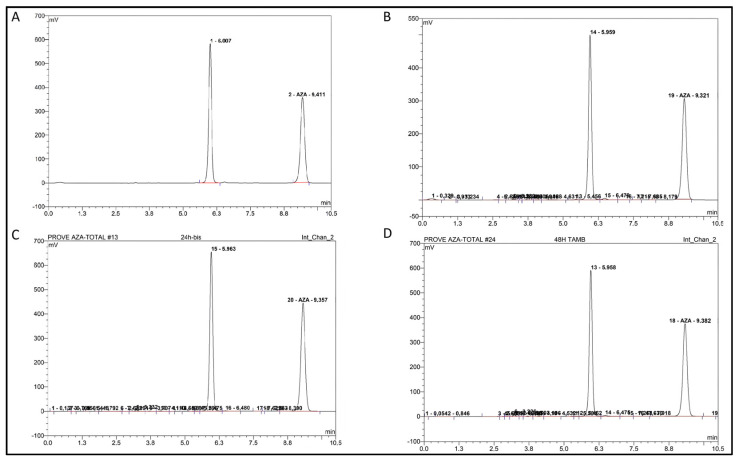
Stability of Azacitidine over time. (**A**): 12 h after reconstitution, (**B**): 14 h after reconstitution, (**C**): 24 h after reconstitution, (**D**): 48 h after reconstitution. The red line beneath the peaks represents the baseline integrated by the chromatographic software, used for accurate peak area integration and quantification.

**Figure 4:**
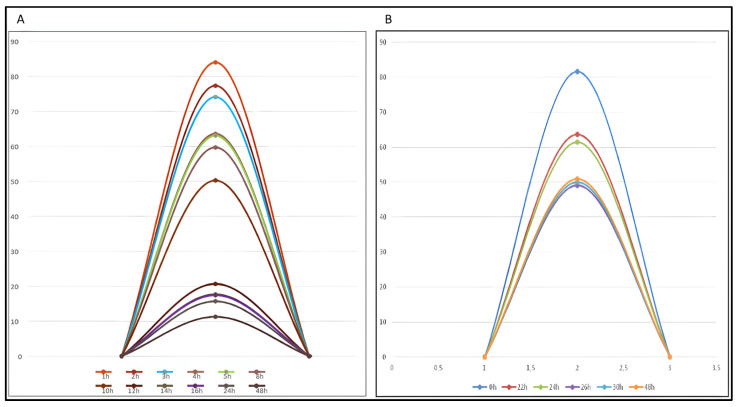
Progressive curve. (**A**): Decrease in reconstituted concentration at 23 °C ± 2 °C, (**B**): Decrease in reconstituted concentration at 4 °C.

Chromatographic analysis revealed new peaks at lower retention times, indicative of hydrolysis products. To visually document the progression of this hydrolytic degradation, Figure 2 presents early- to mid-stage chromatograms (3–10 h), showing the initial decrease of the AZA peak and the emergence of the degradation product. Figure 3 shows the mid- to late-stage progression (12–48 h), where the degradation product becomes the dominant species. Finally, Figure 4 synthesizes these observations into quantitative degradation profiles for both storage temperatures.

**Figure 5:**
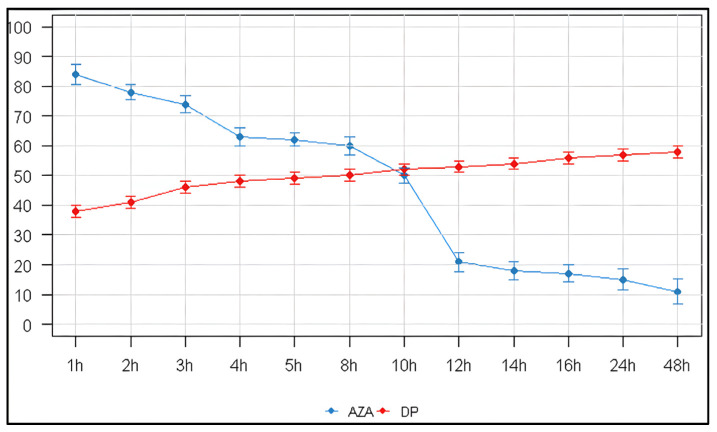
Diagram comparing the concentration of AZA as a function of its degradation product when reconstituted at 23 °C ± 2 °C. AZA: azacitidine, DP: degradation product.

**Figure 6:**
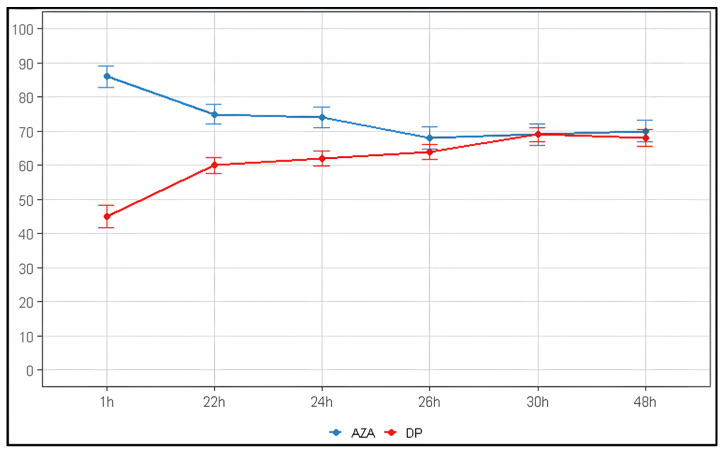
Diagram comparing the concentration of AZA as a function of its degradation product when reconstituted at 4 °C. AZA: azacitidine, DP: degradation product.

The increase in the area of these degradation peaks is inversely correlated with the decrease in the AZA peak area. The relationship between the decreasing AZA concentration and the increasing formation of its main degradation product is visually summarized in Figure 5 (23 ± 2 °C) and Figure 6 (4 °C).

The data obtained for AZA, when reconstituted with water for injection (WFI) at room temperature, show a progressive degradation over time. A marked reduction in concentration is already evident after 4 h, decreasing to 63% ± 2.9%. This indicates that the drug begins to lose integrity earlier than the commonly referenced 8 h threshold. A further decrease to 50% ± 2.5% occurs at 10 h, and by 24 h, the concentration drops to approximately 15% ± 3.5%. When the AZA concentration approaches 50%, the degradation curve enters a plateau phase, during which the levels of AZA and its degradation product become comparable. In parallel, the concentration of the degradation product (DP) increases steadily throughout the observation period. Starting from a measurable level soon after reconstitution, DP rises progressively, reaching about 52% at 10 h and stabilizing around 55–58% from 14 to 48 h. This gradual rise mirrors the decline of AZA and confirms a conversion process in which AZA is progressively degraded into its corresponding product, eventually reaching a state of equilibrium between the parent compound and the degradation product (Figure 5).

Upon reconstitution with WFI preparations stored at 4 °C, the graph shows a proportional trend: the concentration of Vidaza gradually decreases while the DP increases, until approximately 30 h post-reconstitution, at which point the concentrations of both AZA and DP appear to stabilize (Figure 6).

### 2.5. Semi-Quantitative Mass Balance in Azacitidine Degradation

In this study, the primary evidence for the stability-indicating capability of the HPLC method is derived from the mass balance assessment. A semi-quantitative evaluation was performed by monitoring the peak areas of AZA and its main degradation product, DP-6.0. As unequivocally demonstrated in Figure 5 and Figure 6, a clear and consistent inverse correlation exists between the decreasing concentration of AZA and the increasing peak area of DP-6.0. This strong, reproducible correlation serves as the main indicator of method specificity for the primary degradation pathway, providing compelling evidence that DP-6.0 is the major product of AZA hydrolysis under the studied conditions. A precise mass balance (>95%) could not be calculated due to the lack of a reference standard and thus a confirmed response factor for DP-6.0. As LC-MS confirmation was not conducted, the specificity and mass-balance evaluation remain qualitative; therefore, the analytical results should be interpreted as comparative indicators of degradation rather than definitive quantitative measures.

## 3. Discussion

### 3.1. General Discussion of Stability Findings

This study, using model solutions, provides a detailed analysis of the fundamental degradation kinetics of the AZA compound in aqueous solution under controlled conditions. Our findings demonstrate pronounced hydrolytic instability, validating the manufacturer’s recommendation for immediate use [9,19]. While refrigeration provided a statistically significant protective effect compared to room temperature, substantial degradation still occurred within 24 h at 4 °C, indicating that cold storage merely delays rather than prevents potency loss.

While consistent with literature documenting faster degradation at ambient temperature [9], our analysis provides novel quantitative insights, revealing a 4.6-fold difference in degradation rates (−1.64%/hour at 23 °C vs. −0.358%/hour at 4 °C). This precise quantification of the refrigeration benefit represents a significant advancement for clinical practice, where such kinetic parameters can inform more evidence-based handling protocols. The degradation pattern, particularly under refrigeration, appeared non-linear with evidence of a plateau phase. The observed plateau phase in the degradation profile, particularly under refrigerated conditions, strongly suggests a complex kinetic profile beyond simple first-order decay. A plausible mechanistic explanation for this observed stabilization involves the nature of the hydrolytic degradation itself. The initial ring-opening hydrolysis of the triazine ring in AZA to form DP-6.0 is not necessarily a terminal, irreversible step. Under the specific conditions of this study (aqueous solution, neutral pH), the reaction kinetics may become complex, potentially involving a reduction in the net degradation rate as concentrations of reactants and products change. The plateau observed in our study likely represents a point where the overall degradation process slows markedly, which could be consistent with various phenomena, including the formation of a degradation intermediate with higher stability. While we cannot rule out contributions from other factors, the pronounced and consistent nature of the plateau across replicates provides strong evidence for a significant shift in degradation kinetics in the later stages. This observed stabilization behavior adds a crucial, time-dependent perspective to the stability profile of AZA, indicating that its degradation does not follow simple, irreversible first-order kinetics under these conditions.

The observed degradation has direct implications for compliance with pharmacopeial standards. International guidelines (e.g., ICH) typically require that parenteral products remain within 90–110% of the labeled drug content throughout their shelf-life [18]. The ~26% loss of AZA observed after 24 h at 4 °C significantly exceeds this acceptable range, clearly indicating that storage under refrigeration for one day does not maintain pharmacopeial compliance. Even the ~14% loss measured at 1 h post-reconstitution falls outside the strict 10% acceptance limit. This quantitative assessment reinforces that reconstituted AZA cannot be considered stable under refrigeration for pharmaceutically relevant periods. Any delay in administration risks delivering a sub-potent dose, which underscores the non-negotiable need for immediate-use protocols in clinical practice.

Chromatographic analysis revealed the consistent appearance of new peaks at lower retention times, consistent with the formation of more polar degradation products. While comprehensive structural identification was beyond the scope of this study, the observed chromatographic behavior aligns with the known hydrolytic degradation pathway of AZA. The primary degradation product observed at approximately 6.0 min retention time likely corresponds to the open-ring hydrolytic species resulting from triazine ring cleavage, a well-documented transformation for AZA in aqueous solutions [9,19]. The inverse correlation between the decreasing AZA peak and the increasing area of this degradation peak further supports the hydrolytic degradation mechanism.

While this finding requires confirmation through dedicated kinetic studies, it offers valuable insight for future stabilization strategies. Given these complex kinetics, we elected to present the raw degradation data rather than imposing a potentially misleading first-order model.

The rapid degradation has direct clinical implications. Since AZA’s efficacy depends on its structural integrity for DNA incorporation [20], the significant degradation observed under both storage conditions may lead to subtherapeutic exposure if administration is delayed. This not only compromises efficacy but also contributes to substantial pharmaceutical waste [13,14,15]. Furthermore, ensuring chemical stability is a safety concern, as emerging reports of adverse events like AZA-induced lung injury may be influenced by altered drug profiles [21].

Several limitations should be considered. First, using model solutions from pure standard rather than commercial Vidaza^®^ provides fundamental degradation kinetics but limits direct clinical extrapolation. Second, the pharmacological activity of degradation products remains unconfirmed. Finally, regarding the analytical method, while the precision at the lowest concentration level (RSD = 9.76%) exceeded ideal ICH thresholds for absolute quantification, it was deemed acceptable for the primary aim of monitoring relative degradation trends over time. Similarly, the linearity (R^2^ = 0.9928) demonstrated sufficient correlation for the concentration range relevant to this stability study. The method’s core strength for this application lies in its specificity in separating AZA from its main degradation product and its precision at mid-to-high concentration levels (RSD < 5%), which represented the majority of data points in our degradation profiles. While comprehensive forced degradation studies under full ICH conditions are recommended for future method validation, the current method was adequately validated for monitoring hydrolytic degradation trends.

A further consideration regarding the analytical method is the LOQ of 62.8 µg/mL. While this value is higher than those reported in some HPLC methods dedicated to trace analysis or pharmacokinetics, which often achieve LOQs in the low µg/mL or ng/mL range using more sensitive detection (e.g., MS) or extensive sample pre-concentration, it was fully adequate for this stability study. The experimental design utilized a relatively high initial AZA concentration (1 mg/mL = 1000 µg/mL). The LOQ thus represents only 6.3% of the starting concentration, allowing for reliable quantification well into the advanced stages of degradation observed (e.g., down to ~15% remaining at 24 h at room temperature, equivalent to ~150 µg/mL). This LOQ is comparable to, or lower than, those reported in other stability-focused HPLC-UV studies for AZA. For instance, the foundational stability study by Walker et al. reported an LOQ of 50 µg/mL for a 25 mg/mL solution [12], and Iudicello et al. did not explicitly report an LOQ but quantified degradation in a similar high-concentration formulation [9]. Therefore, the sensitivity of the method was fit-for-purpose for monitoring the main degradation trend in this model system, prioritizing robustness and practicality over ultra-trace sensitivity.

This study has some additional analytical limitations that should be acknowledged. Formal system suitability tests, including the analysis of blank injections to document the absence of interference, and peak purity analysis using a diode-array detector (DAD), were not performed. The study focused primarily on exploiting the chromatographic resolution between the parent drug and its degradant to monitor the degradation kinetics. Consequently, the specificity of the method is supported principally by the demonstrated mass balance trend (Figure 5 and Figure 6) and the clear resolution of the peaks, rather than by the full set of recommended validation tests. Future studies will include these verifications to further strengthen the analytical claims.

Building on these findings, future research should prioritize the identification of novel stabilizing agents and formulation strategies. Promising approaches could include lyophilization with alternative excipients, pH adjustment, or the use of protective atmospheres. Such strategies aim to enhance the stability of AZA and ultimately eliminate the requirement for ultra-cold storage. This need is further underscored by the expanding therapeutic role of AZA, as demonstrated by its successful long-term use in novel contexts like maintenance therapy for acute lymphoblastic leukemia post-transplantation [22]. Such emerging treatment paradigms demand reliable and practical storage solutions. Additionally, studies are needed to investigate the impact of extended storage on the pharmacodynamic profile of biologically degraded AZA products. However, the cost-effectiveness and long-term management of such novel formulations will also warrant careful consideration.

The short shelf-life of reconstituted AZA poses particular challenges for hospital pharmacies that must prepare personalized doses extemporaneously. Improving this stability, as achieved with drugs like Bortezomib, would offer significant logistical and economic benefits [23].

In summary, storing AZA at lower temperatures after reconstitution only slightly extends its shelf life and does not prevent its degradation. These findings support current recommendations to use AZA immediately after preparation. Nonetheless, further optimization of preparation and storage protocols could improve drug handling practices and reduce associated costs in oncology care settings. Furthermore, the critical importance of administering the correct, un-degraded dose is highlighted by considering that altered drug profiles from degradation could potentially influence adverse event profiles, though this remains speculative and would require dedicated pharmacovigilance studies for confirmation [21]. Ensuring chemical stability is therefore not only a matter of efficacy and cost but also of patient safety. Looking forward, the recent development and approval of an oral formulation of AZA for maintenance therapy in acute myeloid leukemia [24] could potentially circumvent the stability challenges associated with its injectable counterpart, offering a more convenient and possibly more stable alternative for patients.

### 3.2. Comparison with Previous HPLC Methods

The development of a suitable analytical method is fundamental for accurate stability studies. Several HPLC methods have been reported for the analysis of AZA and its degradation products, each with distinct characteristics tailored to specific objectives, such as formulation development, pharmacokinetics, or stability assessment. To contextualize the methodology employed in the present study, Table 4 provides a comparative overview of key HPLC methods for AZA analysis, including the one developed here, based on parameters such as stationary phase, mobile phase composition, detection wavelength, and their primary merits and limitations.

The method proposed in this work (Entry 1, Table 4) was optimized for monitoring hydrolytic degradation under clinical storage conditions. It employs an isocratic elution with a simple phosphate buffer-acetonitrile mobile phase and a conventional C18 column, ensuring robustness and ease of transfer to quality control laboratories. UV detection at 245 nm provides sufficient sensitivity for the concentration range studied. The main merit of this approach is its practicality and specificity for stability-indicating purposes under the investigated conditions, offering a clear separation of AZA from its main hydrolytic degradant (DP-6.0) without the need for complex instrumentation. Its relative simplicity is also its main limitation, as it does not provide structural confirmation of degradation products, which would require hyphenated techniques like LC-MS.

In comparison, other methods serve different needs. For instance, the method by Iudicello et al. (Entry 2) [9], which also focuses on stability, utilizes a similar C18 column but a different mobile phase system. The earlier method by Walker et al. (Entry 3) [12] established foundational stability data in aqueous solution using a purely aqueous phosphate buffer, and our method builds upon this by introducing a minimal organic modifier to enhance the chromatographic separation for stability-indicating purposes. Methods employing mass spectrometric and NMR detection (Entry 4) [19], while developed for the analog decitabine, offer the most unambiguous structural elucidation of the complex degradation pathways (including anomerization and sugar isomerization) but are less accessible for routine stability testing in many clinical settings. This spectrum of methods, from the foundational to the highly sophisticated, illustrates the evolution of analytical strategies for AZA. This comparison underscores that the choice of analytical method should be aligned with the study’s goals. Our method represents a balanced, fit-for-purpose solution for the quantitative tracking of AZA degradation kinetics in a model system simulating clinical handling, prioritizing robustness and practicality over exhaustive degradant characterization.

The comparative data in Table 5 highlight the temperature-dependent degradation of AZA. Our model solution at 23 °C showed rapid degradation, consistent with the known instability. Direct numerical comparisons of degradation rates between studies should be made with caution due to differing experimental conditions. For instance, under refrigeration (4 °C), the percent remaining at 24 h in our study differs from that reported by Walker et al. [12]. Such differences can be attributed to several factors, including the solution matrix (phosphate buffer vs. sterile water), potential variations in effective pH during degradation, the starting concentration of azacitidine, and the specific analytical method’s capability to resolve and quantify the parent compound amidst degradation products (Table 4). Moreover, while not directly comparable in terms of stability outcomes, the foundational mechanistic work by Rogstad et al. on decitabine [19] elucidates the complex degradation chemistry (e.g., anomerization, sugar isomerization) that underlies the simple loss of parent compound quantified in stability studies like ours, Walker’s, and Iudicello’s. The most striking contrast is observed with the optimized protocol by Iudicello et al. [9], where the use of cold-chain reconstitution and storage between refrigerant gel packs at 4 °C resulted in exceptional stability (>98% remaining at 48 h). This powerful comparison underscores a key conclusion: while refrigeration slows degradation, the stability of reconstituted AZA is critically and favorably influenced by rigorous temperature control from the moment of reconstitution and by advanced storage strategies. Our study defines the ‘baseline’ instability under simple refrigerated storage, whereas the work by Iudicello et al. [9] demonstrates a practical pathway to significantly extend the in-use shelf-life.

### 3.3. Greenness Assessment of the Analytical Method

**Figure 7:**
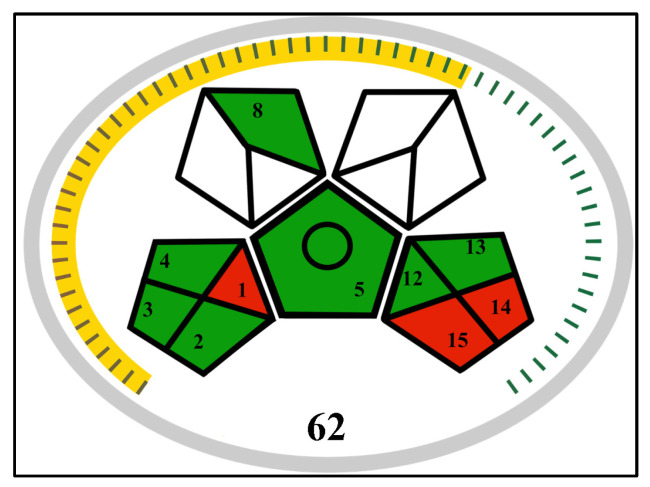
MoGAPI greenness profile of the developed HPLC-UV method. Green indicates low environmental impact, yellow moderate impact, red high impact, while white denotes parameters that are not applicable.

The environmental impact of the developed HPLC-UV method was evaluated using the Modified Green Analytical Procedure Index (MoGAPI) tool, which provides a semi-quantitative score (0–100) and a visual pictogram assessing each step of the analytical procedure. Our method obtained a GAPI score of 62, indicating a moderate greenness profile (Figure 7). The assessment highlighted several favorable aspects: the method requires no sample preparation, uses a low-energy isocratic elution, and operates in a hermetically sealed system, minimizing operator exposure. However, the tool also identified key areas for potential improvement: the generation of more than 10 mL of hazardous waste per analysis and the lack of on-site waste treatment. It is important to note that the “extraction” category in the GAPI tool was marked as “not applicable” for our direct-injection method, which affected the overall score. This evaluation confirms that while the method is practical and robust for stability testing, its environmental footprint is primarily associated with waste generation, a common challenge in conventional HPLC analysis.

## 4. Materials and Methods

### 4.1. Chemicals and Reagents

Azacitidine (AZA) analytical standard (A2385; 5-Azacitidine, ≥98% (HPLC), powder. Sigma-Aldrich: Milan, Italy, 2025. [25]) was used for this study. Although this study used the analytical standard, the instability described is relevant to the commercial product (Vidaza^®^). For the stability assessment, model solutions were prepared from the pure standard to evaluate degradation kinetics under controlled conditions, avoiding potential interference from pharmaceutical excipients. Sterile, pyrogen-free Water for Injections (WFI) was used as the solvent. Dibasic potassium phosphate (K_2_HPO_4_) and 85% orthophosphoric acid (H_3_PO_4_) for HPLC mobile phase preparation were supplied by MP Biomedicals and Fisher Scientific, respectively. All chemicals were of analytical or pharmaceutical grade.

### 4.2. Chromatographic Instrumentation and Conditions

The analysis was performed using a Shimadzu LC-10AD VP HPLC (Shimadzu Corporation, supplied by SpectraLab Scientific Inc., Markham, ON, Canada) system equipped with an autosampler, a column oven, and a Gilson 151 UV/VIS detector (Gilson, Inc., Middleton, WI, USA). Data acquisition and processing were performed using the Chromeleon software (version 7.3.2). Chromatographic separation was achieved using a reversed-phase Hypersil ODS C_18_ column (250 mm × 4.6 mm, 5 μm particle size) (Thermo Scientific, Thermo Fisher Scientific Inc., Waltham, MA, USA). The isocratic mobile phase consisted of a 50 mM potassium phosphate buffer, adjusted to pH 7.0 with orthophosphoric acid and acetonitrile as an organic modifier in a ratio of 98:2. The flow rate was set at 1.0 mL/min, the column temperature was maintained at 25 °C, and the detection wavelength was 245 nm. The injection volume was 20 μL, and the total run time was 10 min. This method was developed to be selective for the separation of AZA from its main degradation products under the studied conditions.

### 4.3. Preparation of Standard Solutions

A stock solution of AZA standard was prepared at a concentration of 1 mg/mL in WFI. This solution was serially diluted with WFI to obtain calibration standards at concentrations of 93.8, 187.5, 375.0, and 750.0 μg/mL. All standard solutions were prepared fresh daily and stored at 4 °C until analysis. A daily calibration curve was constructed by plotting the peak area of AZA against its known concentration. The resulting linear regression equation from this curve was used to calculate the concentration of AZA in the stability samples.

### 4.4. Validation of the HPLC Method

The HPLC method was validated for specificity, linearity, precision, accuracy, LOD, and LOQ according to ICH Q2(R1) guidelines [18] to ensure its suitability for the stability study of AZA. Specificity was assessed by evaluating the separation of AZA from its degradation products. Linearity was determined by analyzing the calibration standards in triplicate. Precision was expressed as the relative standard deviation (RSD%) of six replicate injections of a mid-level standard (250 μg/mL). Accuracy was determined by a recovery study using spiked samples. The LOD and LOQ were determined experimentally using the signal-to-noise ratio approach in accordance with ICH Q2(R1) guidelines [18]. An S/N ratio of approximately 3:1 was used for the LOD and 10:1 for the LOQ, yielding values of 12.56 μg/mL and 62.8 μg/mL, respectively. The results of the validation are reported in Section 2.3.

### 4.5. Sample Preparation for Stability Study

The stability study was conducted on model solutions prepared from the AZA analytical standard at 1 mg/mL in WF to simulate the reconstituted drug. This solution was used directly for the stability testing under the conditions described in Section 4.6. For HPLC analysis at each time point, aliquots from the stability samples were diluted with WFI as needed to fall within the validated calibration range.

The AZA suspension (25 mg/mL) was prepared following routine hospital procedures using sterile refrigerated Water for Injection (2–8 °C), as described in the Vidaza^®^ RCP. The mean pH of the water used was 6.02 ± 0.10, while freshly reconstituted Vidaza^®^ showed a pH of 6.8 ± 0.1 at time zero.

Sampling was performed at defined intervals under two conditions:

- Room temperature (23 ± 2 °C): 0, 1, 2, 3, 4, 5, 8, 10, 12, 14, 16, 24, and 48 h.
- Refrigerated (4 °C): 0, 22, 24, 26, 30, and 48 h. Sampling intervals for the refrigerated condition were chosen based on the practical constraints of laboratory working hours, which limited the feasible times for HPLC analysis.

These experimental parameters were used for the evaluation of AZA stability by HPLC analysis.

### 4.6. Stability Study Protocol

The model solution (1 mg/mL) was divided into aliquots and stored under two conditions: Group A (RT Storage), stored at 23 ± 2 °C. Samples were withdrawn at 0, 1, 2, 3, 4, 5, 8, 10, 12, 14, 16, 24, and 48 h; Group B (Refrigerated Storage), stored at 4 °C. Samples were withdrawn at 0, 22, 24, 26, 30, and 48 h. At each time point, an aliquot was appropriately diluted with WFI to fall within the linear range of the calibration curve and analyzed in duplicate by HPLC. The concentration of AZA in each sample was calculated using the linear regression equation from the daily calibration curve. The stability of AZA at each time point (t) was then expressed as the percentage of the initial concentration (t = 0) using the formula: % Remaining AZA = (Concentration at time t/Concentration at t = 0) × 100.

### 4.7. Preparation of Degraded Samples for Specificity

Forced degradation under thermal stress was performed to confirm the method’s specificity for separating AZA from its main hydrolytic degradation product. An AZA standard solution (1 mg/mL) was stressed by heating at 60 °C for 2 h to accelerate hydrolysis. This degraded solution was analyzed to identify the retention time of the main degradation product and to ensure it was well-separated from the AZA peak.

### 4.8. Statistical Analysis

Linear regression was performed using GraphPad Prism v10 (GraphPad Software, Inc., La Jolla, CA, USA), with degradation rates compared between storage conditions using analysis of covariance (ANCOVA). Data are presented as mean ± standard deviation (SD). A *p*-value of less than 0.05 was considered statistically significant.

## 5. Conclusions

This study developed an HPLC method suitable for evaluating the stability of AZA. While the method’s precision was lower at concentrations near the LOQ (RSD 9.76%), it proved fit-for-purpose for tracking the main degradation trend, including the consistent formation and chromatographic resolution of the primary degradation product (DP-6.0). The findings confirm the inherent instability of the AZA molecule in aqueous solution. A key observation was the plateau phase, indicating a complex degradation profile with a marked reduction in the degradation rate over time. The kinetic data suggest a significant temperature-dependent degradation process. It is crucial to emphasize that these conclusions are derived from a model system at 1 mg/mL. Therefore, they should be interpreted as a proof-of-concept and a foundation for further research. The insights gained here, particularly the identification of the main degradation pathway and the observed stabilization phase, warrant confirmation through a comprehensive stability study conducted on the commercial pharmaceutical formulation under standardized conditions. Ultimately, optimizing the storage of AZA based on robust, clinically relevant data could lead to improved efficiency and reduced waste in oncology care. Further kinetic and formulation studies are warranted to extend the stability of reconstituted AZA.

## Figures and Tables

**Table 1:** Summary of HPLC method validation parameters for the determination of Azacitidine.

Parameter	Result
Linearity range	93.8–750.0 μg/mL
Correlation coefficient (R^2^)	0.9928
Precision (RSD%)	
Mid/High Concentration	<5%
Near LOQ	9.76%
Accuracy (Mean Recovery)	96%
LOD	12.56 μg/mL
LOQ	62.8 μg/mL

**Table 2:** Stability of azacitidine (1 mg/mL) stored at Room Temperature (23 ± 2 °C).

Time (h)	Remaining AZA (%)	95% CI
1	84 ± 3.3	82.5–85.5
2	78 ± 2.5	75.6–80.4
3	74 ± 2.8	70.6–77.4
4	63 ± 2.9	59.2–66.8
5	62 ± 2.2	58.0–66.0
8	60 ± 3.1	55.9–64.1
10	50 ± 2.5	45.0–55.0
12	21 ± 3.2	13.6–28.4
14	18 ± 3.0	11.0–25.0
16	17 ± 2.9	10.1–23.9
24	15 ± 3.5	7.9–22.1
48	11 ± 4.1	2.9–19.1

**Table 3:** Stability of azacitidine (1 mg/mL) stored under Refrigeration (4 °C).

Time (h)	Remaining AZA (%)	95% CI
1	86 ± 3.1	79.9–92.1
22	75 ± 2.8	69.5–80.5
24	74 ± 3.0	68.1–79.9
26	68 ± 3.2	61.7–74.3
30	69 ± 3.1	62.9–75.1
48	70 ± 3.2	63.7–76.3

**Table 4:** Comparison of HPLC methods for the analysis of azacitidine (AZA) and its degradation products.

Entry	Method Focus/Source	Stationary Phase	Mobile Phase (*v*/*v*)	Detection (λ/Technique)	Key Merits	Main Limitations/Notes
1	This work (Stability study)	Hypersil ODS C_18_ (250 × 4.6 mm, 5 µm)	50 mM Potassium Phosphate Buffer (pH 7.0): ACN = 98:2 (Isocratic)	UV, 245 nm	Optimized for stability-indicating assay. Simple, robust, and practical for clinical setting monitoring. Good separation of AZA from the main hydrolytic degradant (DP-6.0).	No structural confirmation. UV is less specific than MS for unknown degradants.
2	Iudicello et al., 2021 [9] (Formulation stability)	Ascentis Express C18 (150 × 4.6 mm, 2.7 µm)	Gradient: 0.02 M Ammonium Acetate Buffer (pH 6.9): MeOH: ACN	UV, 210 nm	Validated per ICH. Used for the stability of an optimized cold-chain suspension. Enables identification of multiple degradants (RGU, RGU-CHO).	Gradient method, more complex setup. Uses MS (HRMS) for structural confirmation of degradants.
3	Walker et al., 2012 [12] (Stability in SWFI)	Waters Nova Pak C18 (300 × 3.9 mm, 5 µm)	0.05 M Potassium Phosphate Buffer (pH 7.0) (Isocratic)	UV, 245 nm	Foundational stability method. Validated for AZA in sterile water. Established baseline degradation kinetics at various temperatures.	Isocratic, simple. Lacks detailed degradant profiling. Uses phosphate buffer (pH 7).
4	Rogstad et al., 2009 [19] (Degradation characterization)	C-18 Analytical Column (Supelco, 25 cm × 4.6 mm, 5 µm)	20 mM Ammonium Acetate (pH 6.8) (Isocratic)	UV (PDA) and QTOF/MS	Exceptional separation of >9 degradation products of decitabine (DAC). Structural identification via MS and NMR. Reveals complexity (anomers, sugar isomers).	Method developed for decitabine, a closely related structural analog. The complexity and use of MS make it more suited for mechanistic research than routine monitoring.

ACN: Acetonitrile; MeOH: Methanol; SWFI: Sterile Water for Injection.

**Table 5:** Comparative overview of azacitidine degradation under different storage conditions as reported in key stability studies.

Study/Formulation	Storage Condition	Concentration	Key Stability Findings (% Remaining, % Loss)	Primary Analytical Method	Ref.
This study (Model solution)	Room Temp. (23 ± 2 °C)	1 mg/mL	15% remaining at 24 h (>85% loss). ~50% loss by 10 h	HPLC-UV (C18; 245 nm)	-
	Refrigeration (4 °C)	1 mg/mL	74% remaining at 24 h (~26% loss). Degradation rate 4.6× slower than RT		
Walker et al., 2012 (Sterile water for injection)	Room Temp. (23 °C)	25 mg/mL	~ 84.3% remaining at 9.6 h (~15.7% loss)	HPLC-UV	[12]
	Refrigeration (4 °C)	25 mg/mL	93.6% remaining at 24 h (~6.4% loss). 71.4% remaining at 96 h (~28.6% loss)		
Iudicello et al., 2021 (Optimized suspension formulation)	Refrigeration (4 °C)	25 mg/mL	>98% remaining at 48 h (<2% loss). ~95.2% remaining at 96 h (~4.8% loss)	HPLC-UV	[9]
Rogstad et al., 2009 (Decitabine, model solution) *	37 °C, pH 7.4 (Physiological conditions)	~9 mM	Half-life of β-decitabine: ~10.1 h. Formation of a complex profile of >9 degradation products (anomers, sugar isomers). Defines pathway complexity	HPLC-UV/MS, NMR	[19]

Explanatory note: Differences in absolute degradation rates between studies (e.g., at 4 °C) can be attributed to various experimental factors, including: the solution matrix (buffer vs. water vs. excipient-containing formulation), the effective pH during degradation (influenced by concentration and buffer capacity), the starting concentration of azacitidine, and the specificity of the analytical method used to quantify the parent compound (see Table 4). Despite these differences, all studies converge in confirming the intrinsic hydrolytic instability of azacitidine and the significant protective effect of refrigeration. * The study by Rogstad et al. is included for its fundamental contribution to understanding the complex degradation pathway of aza-nucleosides, although conducted on decitabine (DAC) under different conditions (37 °C). RT: Room Temperature.

## Data Availability

The original contributions presented in this study are included in the article. Further inquiries can be directed to the corresponding authors.

## Abbreviations

The following abbreviations are used in this manuscript:

AZA	Azacitidine
HPLC	High-Performance Liquid Chromatography
LC-MS	Liquid Chromatography-Mass Spectrometry
WFI	Water for Injection
